# Partial Melting of Subducted Sediments Produced Early Mesozoic Calc-alkaline Lamprophyres from Northern Guangxi Province, South China

**DOI:** 10.1038/s41598-017-05228-w

**Published:** 2017-07-07

**Authors:** Hui-Min Su, Shao-Yong Jiang, Dong-Yang Zhang, Xiang-Ke Wu

**Affiliations:** 1 State Key Laboratory of Geological Processes and Mineral Resources, Collaborative Innovation Center for Exploration of Strategic Mineral Resources, Faculty of Earth Resources, China University of Geosciences, Wuhan, 430074 China; 2 State Key Laboratory for Mineral Deposits Research, Department of Earth Sciences, Nanjing University, Nanjing, 210093 China; 3 Geological Survey Institute of Guangxi, Guangxi Bureau of Geology and Mineral Prospecting and Exploitation, Nanning, 530023 China

## Abstract

There is growing agreement that subducted sediments recycled into the deep mantle could make a significant contribution to the generation of various mantle-derived rocks. However, solid evidence and examples to support this view are few, and whether or not the subducted sediments can act as the dominating material source for the magma is unclear. Here, we report a comprehensive geochemical study that demonstrates that the newly identified Early Mesozoic calc-alkaline lamprophyres in the northern Guangxi Province, southeastern Yangtze Block in South China were likely derived in large part from the partial melting of the subducted terrigenous sediments in the deep mantle. The investigated lamprophyres are SiO_2_-rich minettes, characterized by moderate TFeO and MgO and high Mg^#^ (>70). The multi-element pattern shows a typical crustal-like signature, such as enrichments in large-ion lithophile elements (LILE) and light rare earth elements (LREE) with troughs in Nb-Ta, Ti and Eu and peaks in Th-U and Pb. These rocks also show sediment-like ratios of Nb/U, Nb/Th and Ce/Pb, together with extremely radiogenic ^87^Sr/^86^Sr (0.71499–0.71919), unradiogenic ^143^Nd/^144^Nd (0.51188–0.51195) and radiogenic ^207^Pb/^204^Pb (15.701–15.718) isotopic compositions.

## Introduction

Calc-alkaline lamprophyres are melanocratic hypabyssal igneous rocks that generally form dykes and sills and that are characterized by a panidiomorphic porphyritic texture carrying hydrous mafic phenocrysts. Lamprophyres are widely considered to represent primary mantle-derived magmas and are characterized by extreme enrichment in incompatible elements and radiogenic isotopes that typically reflect a crustal source^1, 2^. A typical model for the genesis of lamprophyre suggests a metasomatized subcontinental lithospheric mantle source that has been enriched by aqueous fluids and/or melts of subducted slab/sediments during an ancient event^3–5^.

Abundant geological and geophysical observations of sediment subduction suggest that most crustal material has been transported by the subducted channel into the mantle^6, 7^. Recently, a close correspondence between the geochemical and isotopic signature of Mediterranean orogenic lamproites, the sediments in the trench, and the sedimentary wedge was reported by Prelević *et al*.^8^. This correspondence indicates that the subducted sediments could have been introduced into the mantle and may be responsible for the abnormal trace-element and isotopic signatures of these mantle-derived rocks. However, whether the subducted sediments play a major role, or if they only partially contribute via fluid or melt metasomatism to the primary mantle magma source, is still unknown.

Several Early Mesozoic lamprophyre dykes were recently identified in the northern Guangxi Province, southeastern Yangtze Block (see Supplementary Geological Setting and Petrography). The extreme isotopic signatures and chemical element enrichment exhibited in these lamprophyres demonstrate obvious fingerprints of sedimentary materials, implying that subducted sediments play a dominant role as a mantle source.

## Results

The lamprophyre samples studied in this paper were collected from the Danyang and Niulangpo districts in the Sanjiang-Rongshui region (Supplementary Figure 1). The Danyang samples contain predominantly mica and subordinate clinopyroxene phenocrysts, but the Niulangpo samples are characterized by the presence of mica as the only mineral in the phenocryst phase (Supplementary Figure 2).

### Ar-Ar dating

**Figure 1:**
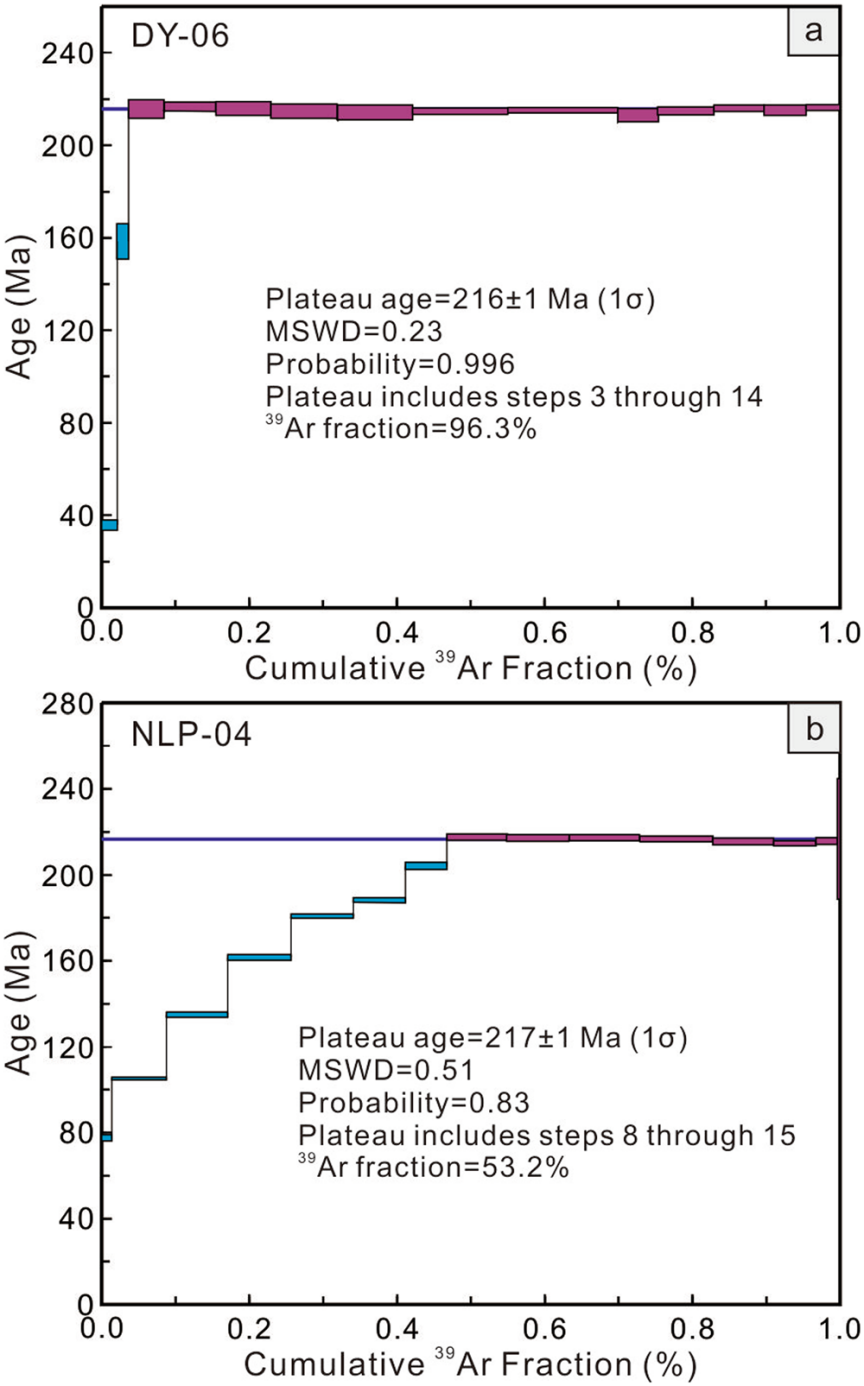
^40^Ar-^39^Ar plateau ages for phlogopite from the Danyang and Niulangpo lamprophyres.

Two phlogopite separates from the Danyang (DY-06; 109°22′54″E, 25°25′45″N) and Niulangpo lamprophyres (NLP-04; 109°35′45″E, 25°38′48″N) were collected for Ar-Ar dating, yielding plateau ages of 216 ± 1 Ma and 217 ± 1 Ma (Fig. 1 and Supplementary Table 1). These data suggest that the investigated lamprophyres were formed during the Early Mesozoic.

### Major and trace elements

The lamprophyres are characterized by elevated SiO_2_ content (53.80–56.92 wt.%), uniformly moderate TFe_2_O_3_ (5.11–5.74 wt.%) and MgO (6.03–7.51 wt.%) content, and high Mg# (>70) (Supplementary Table 2). The Danyang samples are generally enriched in Al_2_O_3_ and K_2_O with values ranging from 12.92–14.10 wt.% and 5.04–6.57 wt.%, whereas in Niulangpo samples, these oxides are less than 12 wt.% and 3.5 wt.%, respectively. Although low in TiO_2_ and CaO, the Danyang lamprophyres show relatively lower values (<1 wt.% and<5 wt.%) than the Niulangpo rocks (>1 wt.% and>5 wt.%). The Niulangpo lamprophyres show a high-K calc-alkaline characteristic, while the Danyang samples display a shoshonitic nature (Supplementary Figure 3).

**Figure 2:**
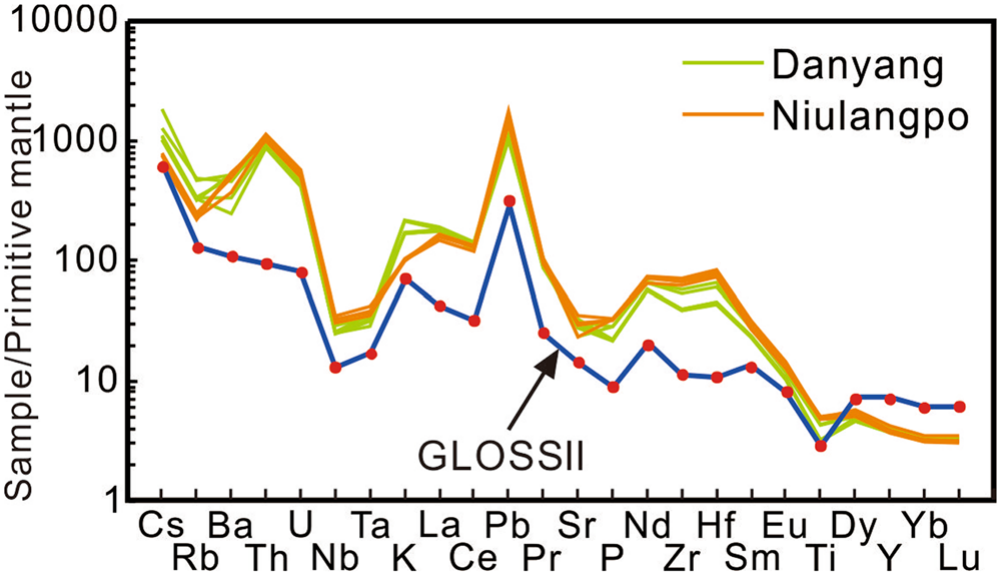
Primitive mantle normalized multi-element variation diagram of the investigated lamprophyres. The normalization values for primitive mantle are from Taylor and McLennan^49^. The composition of the global average of subducted sediment (GLOSS II) is from Plank^9^.

The studied lamprophyres have high but discrepant compatible elements. Specifically, Cr and Ni content in the Niulangpo samples (396–440 ppm and 247–381 ppm) are significantly higher than those in the Danyang samples (247–347 ppm and 179–283 ppm). The Danyang samples have higher Rb (203–308 ppm versus 140–159 ppm) but noticeably lower Zr (433–646 ppm versus 699–799 ppm) and Hf (13–20 ppm versus 23–26 ppm) compared to the Niulangpo lamprophyres. Plotting the values on a multi-element variation diagram, the Danyang and Niulangpo lamprophyres exhibit broadly similar patterns (Fig. 2). They are significantly enriched in large-ion lithophile elements (LILE, e.g., Ba, Rb and K) relative to highfield-strength elements (HFSE, e.g., Nb, Ta and Ti). Sharp troughs at Nb-Ta and Ti and small troughs at Sr and P are present, whereas a peak is evident at Pb. In general, the incompatible elements exhibit great similarity to GLOSS II (Global subducting sediments; ref. 9). However, the lamprophyres studied show a higher abundance of Th, U, Pb and LREE with respect to GLOSS II (Fig. 2).

### Sr-Nd-Pb isotopes

**Figure 3:**
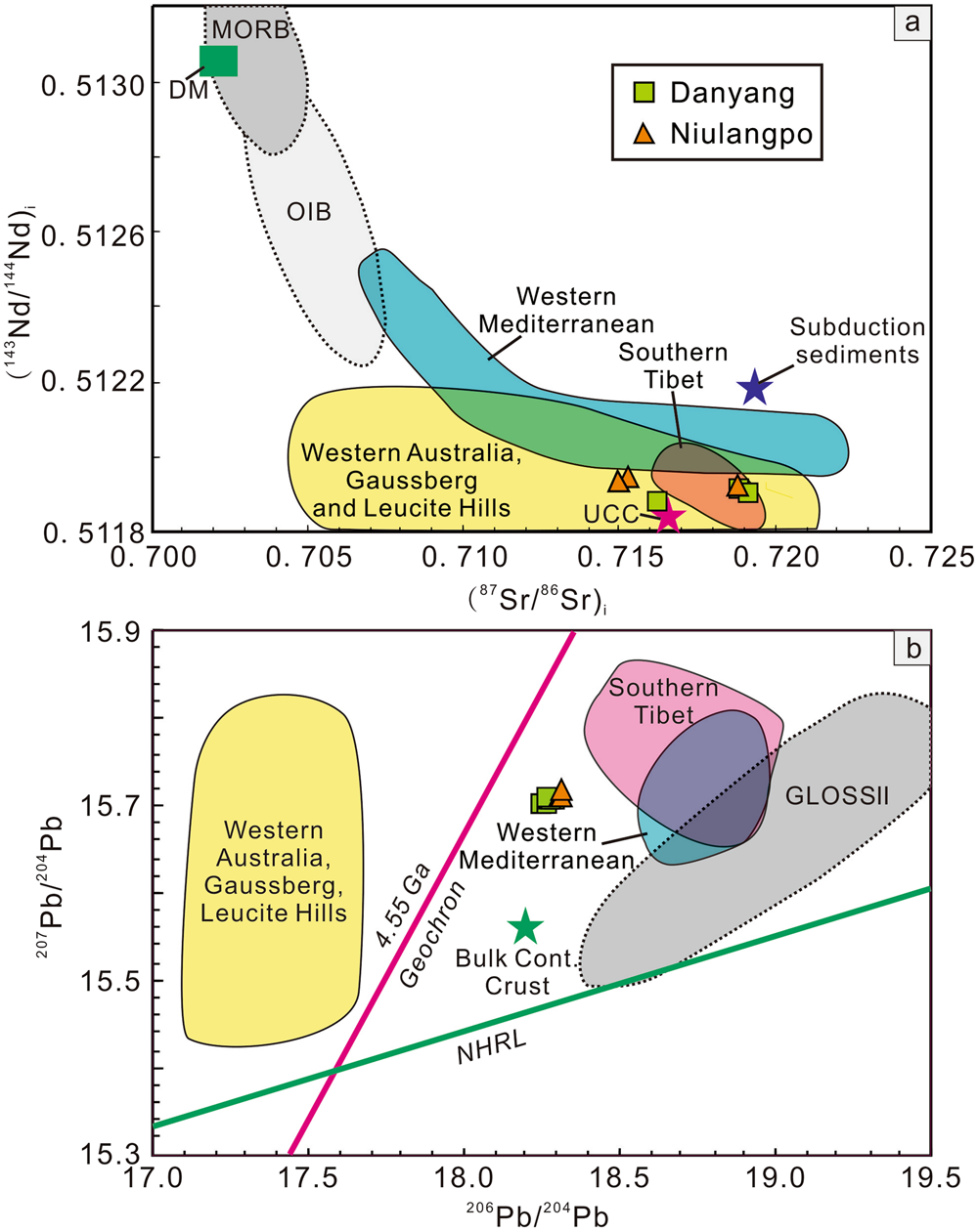
Plot of (^143^Nd/^144^Nd)_i_ vs. (^87^Sr/^86^Sr)_i_ and ^207^Pb/^204^Pb vs. ^206^Pb/^204^Pb for the studied lamprophyres. Data sources: Western Mediterranean lamproites^11^, Southern Tibet potassic rocks^10^, lamproites from Western Australia, Gaussberg and Leucite Hills^12–14, 50, 51^, GLOSS II^9^, UCC (Upper Continental Crust)^52^, Subduction sediments^53^, Bulk Cont. Crust (Bulk Continental Crust)^32^, NHRL (Northern Hemisphere reference line) and 4.55 Ga geochron^15^.

Both whole rock and phlogopite separates were analyzed for Sr and Nd isotopic compositions. Despite the variable and extremely high ^87^Sr/^86^Sr values, the calculated initial ^87^Sr/^86^Sr ratios in whole rock and phlogopite separates from Danyang and Niulangpo are indistinguishable and within the analytic error (Supplementary Table 3). Generally, the Danyang and Niulangpo lamprophyres are characterized by significantly high initial ^87^Sr/^86^Sr (0.71628–0.71919 and 0.71499–0.71881) and low initial ^143^Nd/^144^Nd (0.51188–0.51191 and 0.51192–0.51195) values. Their Sr-Nd isotopic compositions are within the range of those reported for the potassic volcanic rocks in southern Tibet and the lamproites in the western Mediterranean, western Australia, Leucite Hills and Gaussberg (Fig. 3a)^10–14^.

The measured Pb isotope composition of the calc-alkaline lamprophyres exhibit relatively restricted variations with ^206^Pb/^204^Pb = 18.253–18.316, ^207^Pb/^204^Pb = 15.701–15.718, and ^208^Pb/^204^Pb = 38.652–38.694. When plotted on the ^207^Pb/^204^Pb vs. ^206^Pb/^204^Pb diagram (Fig. 3b), all samples fall to the right of the 4.55 Ga Geochron and significantly above the Northern Hemisphere Reference Line (NHRL, ref. 15). Compared to the lamproites in the western Mediterranean and potassic volcanic rocks in southern Tibet, they show similar ^207^Pb/^204^Pb ratios but lower ^206^Pb/^204^Pb ratios (Supplementary Table 4) and plot above the GLOSS II field (Fig. 3b).

### Mineral compositions of mica

Mica from both lamprophyres shows phlogopite to biotite composition (Supplementary Table 5). In detail, the large inner part of the mica crystal (core) is phlogopite, whereas a narrow rim shows the composition ranging from phlogopite to biotite (Supplementary Figure 2). For convenience, hereinafter we refer to these phenocrysts as phlogopite. As a corollary, there are striking differences in MgO and FeO content in the cores compared to the rims. In the Dangyang lamprophyres, MgO concentrations in the cores and rims are 19.90–20.91 wt.% and 10.91–18.96 wt.%, respectively; and FeO concentrations are 6.42–8.65 wt.% and 9.36–17.92 wt.%, respectively. Similar trend occurs in the Niulangpo phlogopite phenocrysts (MgO: 17.82–22.47 wt.% vs. 8.70–17.54 wt.%; FeO: 5.43–10.29 wt.% vs. 10.63–20.34 wt.%). Mg^#^ values of the phlogopite phenocrysts from both Danyang and Niulangpo decrease rimward. The compositional trend from a high Mg and Si core to a low Fe, Al, Ti and Mn rim is very common.

## Discussion

The Danyang and Niulangpo lamprophyres are calc-alkaline minettes (see Supplementary Geological Setting and Petrography). They may originate from broadly similar source materials with similar processes as demonstrated by their similarity in both trace element and isotopic characteristics. All are characterized by primitive composition of Mg# (>70), Ni (>175 ppm) and Cr (>245 ppm), and low Nb/La ratios (<0.25). These features clearly represent relatively primary mantle melts which have experienced insignificant fractionation (see Supplementary Discussion). However, multi-element patterns (enrichments in LILE and LREE) and Sr-Nd isotopic characteristics require an “enriched” component in the source. Two possible explanations for the origin of the investigated lamprophyres are discussed below.

Conventionally, lamprophyric rocks with these characteristics are interpreted as being derived from an enriched lithospheric mantle source which had been metasomatized prior to partial melting^16–18^. The high K_2_O contents and significant LILE enrichment in such lamprophyric rocks are believed to indicate the existence of a potassium-rich mineral phase (phlogopite or amphibole) in the mantle source region^19^. Moreover, the extreme radiogenic isotope signatures of the enrichments in the source of lamprophyres usually have been explained as being due to either the antiquity of the metasomatic event or crustal contamination of the lithospheric mantle during more recent subduction^3, 11, 20–22^. If a domain were metasomatized by antecedent melt/fluid, then preservation of such heterogeneity would require temperatures to decrease to subsolidus conditions and remain so until the next magmatic episode. Any thermal perturbations or lithospheric thinning would result in partial melting of the geochemically anomalous domains^23^. The lithosphere mantle beneath the South China Block experienced metasomatism during the Neoproterozoic subduction related to the collision between the Yangtze and Cathaysia blocks, or the so-called Jiangnan Orogeny^24^. However, considering the complex Phanerozoic tectonic and magmatic evolution history of South China generally associated with the extensive Caledonian and Indosinian Orogeny, the Precambrian metasomatized domain was less likely to have remained chemically isolated until the early Mesozoic. Moreover, metasomatism can be envisioned as the interaction between a LILE-enriched fluid and ambient (often depleted) mantle peridotite. Stalder *et al*.^25^ showed experimentally that LILE (e.g., Sr, Ba) is more soluble in high P-T aqueous fluids than the REE. Thus, high Sr/Nd (>17) and Ba/La (>40) ratios are expected to be observed if an aqueous fluid component was added to the mantle sources^10, 26^, which is inconsistent with the low Sr/Nd (5–9) and Ba/La (14–37) ratios observed in the lamprophyres studied herein. Admittedly, metasomatism by partial melting of isotopically evolved sediments in the lithospheric mantle could be responsible for the radiogenic isotope signatures and associated trace element ratios; whereby a mixing trend of two/multiple end-members could usually be recognized by the Sr-Nd-Pb isotopes in this case^10, 11^. Thus, we discount the importance of a metasomatized lithosphere mantle in the origin of the lamprophyres in this study, although the possibility cannot be ruled out completely.

Alternatively, we propose a relatively brief model and argue that partial melting of a subducted sediment component can account for the trace element and isotopic compositions, and their associated ratios. Several lines of evidence support this proposal.

First, a crust-derived subducted sediment component could be easily recognized on the basis of the current major and trace element data. Like modern subduction sediments, the studied lamprophyres show “jagged” spidergrams, marked by the presence of troughs in Nb-Ta, Ti and Eu, and remarkable peaks in Th-U and Pb (Fig. 2). The ratios of Th/U (8.02–8.78), Nb/Ta (12.52–15.37) and Ce/Pb (1.89–3.09) are also typical for evolved continental crust materials. The Hf/Sm ratio is a proxy for sediment type (terrigenous or pelagic sediments), and the high Hf/Sm ratios (1.31–1.89) in the Danyang and Niulangpo samples further demonstrate the importance of the subducted terrigenous sediments.

**Figure 4:**
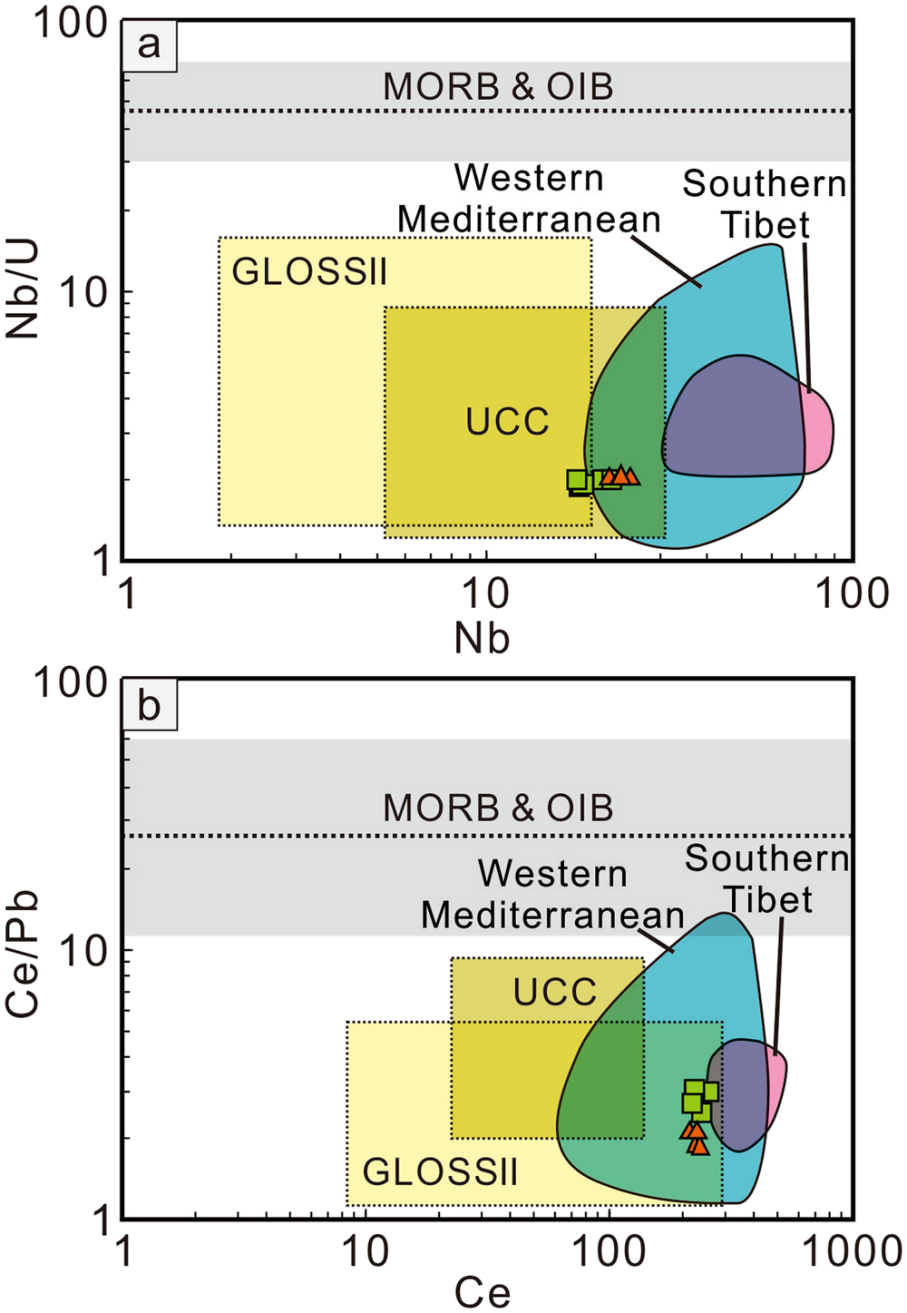
Diagrams of Nb/U vs. Nb (**a**) and Ce/Pb vs. Ce (**b**) for the Danyang and Niulangpo lamprophyres. Data Sources: Western Mediterranean lamproites^11^, Southern Tibet potassic rocks^10^, MORB and OIB^27, 28, 54–57^, and UCC^58–60^.

Second, the depletion of Nb relative to U and Th, and Ce relative to Pb in subducted sediments leads to significantly lower ratios of Nb/U, Nb/Th and Ce/Pb compared to the relatively high and near constant values found in MORB and OIB^27, 28^. Thus, these ratios have been widely used as tracers to detect recycled crustal material in mantle-derived melts^29^. The Danyang and Niulangpo lamprophyres have markedly low Nb/U and Ce/Pb ratios that fall within or below the field of subduction sediments and upper continental crust, suggesting that they were derived from sediment-rich sources (Fig. 4a,b).

Finally, Pb isotopes are the most sensitive indicators of contamination in the mantle by crust-derived sediments, because of the extreme difference in concentrations between Pb in sediments (21.2 ppm, GLOSS II; ref. 9) and Pb in primitive mantle (0.185 ppm; ref. 30). The high ^207^Pb/^204^Pb ratios, along with the extremely radiogenic Sr and unradiogenic Nd isotopes, observed in the Danyang and Niulangpo samples, are also consistent with a significant contribution of sediment to their sources. In several young volcanic provinces, such as in the Mediterranean, Southern Tibet and Western Samoa, an increasing amount of evidence shows a contribution of crust-derived sediments to the mantle sources of the lamproitic and basaltic rocks^10, 11, 31^. Nevertheless, these mantle-derived mafic rocks usually show variable isotopic compositions, which have been explained as a mixing of subordinate sediments and dominant melts from other geochemical reservoirs (e.g., primitive or depleted mantle). For instance, the proportion of recycled sediment in the mantle source of the Samoan lava was estimated to be no more than 5% according to a relevant simulated calculation^31^. However, in our study, the isotopic compositions of the lamprophyres show fairly consistent Pb, Nd and Sr isotope ratios, with no discernible trends seen in the (^143^Nd/^144^Nd)_i_ vs (^87^Sr/^88^Sr)_i_ and ^207^Pb/^204^Pb vs ^206^Pb/^204^Pb diagrams (Fig. 3a,b). Therefore, these isotope data represent a single source/melt rather than a mixture. Thus, the studied lamprophyres were likely derived from a source containing a dominant proportion of partial melts from sediments, resulting in the extreme isotope signatures. Due to the strong age sensitivity of the U-Pb isotope system, lamprophyre Pb isotopic composition could be significantly affected by the isolation age of the possible sources. Any ancient metasomatic/isolation episode would cause significant retardation of ^206^Pb/^204^Pb ratios, causing their isotopic evolution to plot left of the Geochron. For instance, the lamproites from Western Mediterranean and potassic rocks from Southern Tibet are interpreted to be derived from a mantle source contaminated with continental-derived sediments which were subducted throughout the relatively recent closure of the Tethys Ocean. They plot to the right of the Geochron in a position very close to the field of modern subduction sediments (Fig. 3b). By contrast, the derivation of the lamproites from western Australia, Leucite Hills and Gaussberg, which plot to the left of the Geochron (Fig. 3b), are interpreted as being the melting products of sediments that were isolated for more than 2 Ga. The lamprophyres in this study display ^206^Pb/^204^Pb ratios analogous to the bulk continental crust^32^. As seen in the ^207^Pb/^204^Pb vs ^206^Pb/^204^Pb diagram, the investigated samples show great similarity to both the lamproites from Western Mediterranean and potassic rocks from Southern Tibet, and plot to the right of the Geochron. Moreover, all of the samples plot within or near the field of GLOSS II. Thus, the Pb isotopic signature in the studied samples is more likely to be introduced to the mantle during more recent subduction and thus could be entirely accounted for by inheritance from the subducted sediments.

There is a growing agreement about the presence of recycled sediment in the mantle, although clear examples are still few^10, 11, 31, 33^. Prelević *et al*.^11^ proposed that the crustal component stored within the lithospheric mantle of Alpine-Himalayan belt is ubiquitously present, and contaminated the mantle sources of the regional lamproitic rocks. Compositionally heterogeneous sediments are known to be introduced into a primitive (often depleted) mantle by subduction. The subduction of the paleo-Pacific plate beneath the Chinese continent throughout the Phanerozoic history of the South China Block is the process that most probably provided sediments for the origin of the investigated lamprophyres, although the timing of the initial subduction is still controversial^34–37^. Global and regional tomography shows that most of the slab materials under the Western Pacific and Eastern China have been subducted down to a great depth and have become stagnant in the mantle transition zone under China^38, 39^. Furthermore, the upwelling of hot asthenospheric materials associated with the deep subduction and stagnancy of the Pacific slab caused the intraplate volcanism in Northeastern China^40^. Similarly, the paleo-Pacific plate beneath South China could have subducted down to great interior depths in the early Mesozoic^35^ and supplied the crust materials for formation of the lamprophyres studied.

**Figure 5:**
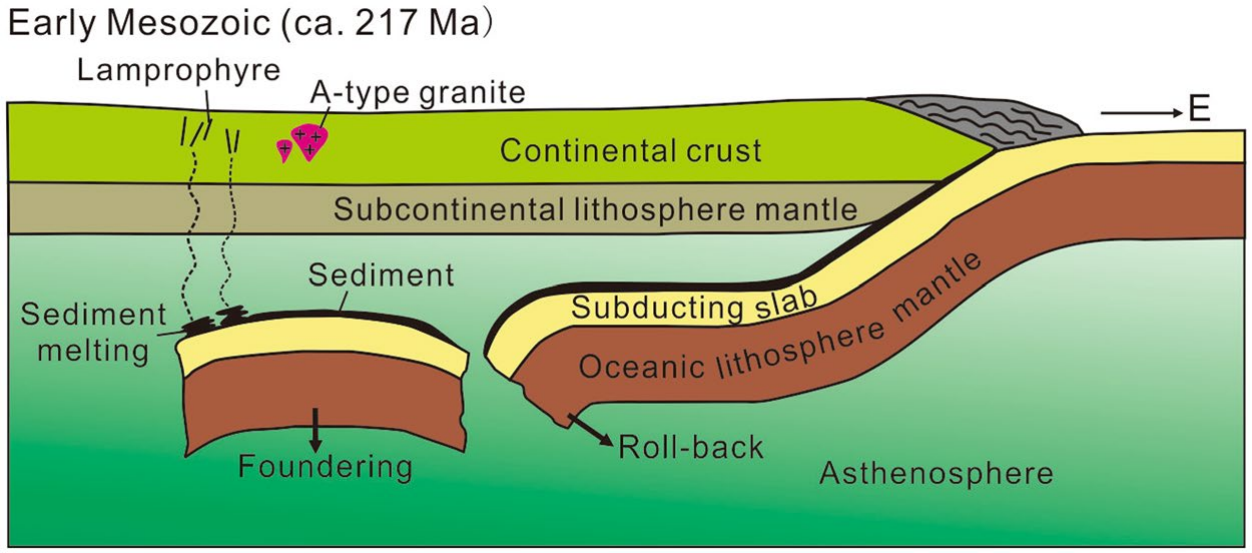
A simplified model showing the magma processes of the Early Mesozoic lamprophyres in Northern Guangxi Province.

It is suggested that the South China Block was dominated by an extension regime in the Late Triassic due to the occurrence of A-type granites and the absence of compressive deformation structures^41–44^. To explain the tectonic evolution processes and associated magmatism of South China during the Mesozoic, several models have been proposed, including models involving continental collision between the South China and Indosinian blocks^37, 45, 46^, and models involving the subduction of the paleo-Pacific Plate^35, 47^. The geochemical evidence from the lamprophyres in this study clearly points to the presence of subducted sediments in the mantle of the South China Block during the Early Mesozoic, providing new geological support for the flat-slab subduction and slab foundering model proposed by Li and Li^35^. Specifically, the paleo-Pacific Plate could have arrived at the northern Guangxi as early as the Late Triassic at ~217 Ma. Following the break-off and foundering event of the subducting/subducted flat slab, the sediments above the slab generated partial melts which variably interacted with the overlying depleted mantle, giving rise to the lamprophyric magma (Fig. 5).

## Conclusions

Several Early Mesozoic lamprophyre dykes were recently discovered in the northern Guangxi Province, southeastern Yangtze Block, South China. The crust-like trace element and isotopic signatures of the investigated lamprophyres suggest that a recycled terrigenous sediment component could have played a major role in their source. The results point to the presence of subducted sediments in the mantle of the South China Block, as early as in the Early Mesozoic. The subducted slab of paleo-Pacific Plate could have probably supplied continental-derived sediment components for the lamprophyres.

## Methods

### Ar-Ar dating

Phlogopite samples were irradiated for 24 h in the nuclear reactor at the Chinese Institute of Atomic Energy in Beijing, with integrated neutron flux at 2.2464 × 10^18^ ncm^−2^. The monitor samples used in this work include the internal standard biotite ZBH-25 (132.7 Ma), Bern 4 M (18.6 Ma), and FCs (28.2 Ma), which were all irradiated. The correction factors for the interfering isotopes produced during irradiation were determined by analysis of irradiated pure materials of K_2_SO_4_ and CaF_2_, yielding the following ratios: (^36^Ar/^37^Ar)_Ca_ = 0.000271, (^39^Ar/^37^Ar)_Ca_ = 0.000652, and (^40^Ar/^39^Ar)_K_ = 0.00703. The samples and monitors were heated at temperatures of 600–1500 °C in a graphite furnace; the heating-extractions included 10–16 steps with each temperature increment of 20 min and purification increment of 25 min. The analysis was performed at the Key Laboratory of Orogenic Belts and Crustal Evolution (Peking University), Ministry of Education, using an RGA 10 mass spectrometer. Measured isotopic ratios were corrected for mass discrimination, atmospheric Ar, blanks, and irradiation-induced mass interference.

### Electron microprobe analysis

Chemical composition analyses of mica phenocrysts were obtained from polished thin sections using a JEOL JXA-8230 electron microprobe analyzer (EMPA) at Key Laboratory of Metallogeny and Mineral Assessment, Chinese Academy of Geological Sciences. Operating conditions were set at 20 kV accelerating voltage, 20 nA beam current, and 5 µm beam diameter. Natural and synthetic materials were used for standardization (Si, Al and Na: Jade, K: K-feldspar, Mg: Mg-olivine, Ca: wollastonite, Fe: magnetite, P: apatite, Ti: rutile, Mn: MnTiO_3_, Cr: Cr_2_O_3_, Ni: NiO). All data were corrected with standard ZAF correction procedures. The accuracy of the reported values for the analyses is 1% to 5% depending on the abundance of the element.

### Major and trace element analysis

Major oxides were analyzed by X-ray fluorescence spectrometer (XRF, 3050E) using fused glass disks at the National Research Center of Geoanalysis, Chinese Academy of Geosciences, with analytical uncertainties ranging from 0.5% to 1.5%. Trace element concentrations were analyzed using an Agilent 7500a inductively coupled plasma mass spectrometer (ICP-MS) at the Institute of Geology and Geophysics (ICG), Chinese Academy of Sciences. Precisions were generally better than 5% for most elements.

### Sr-Nd-Pb isotope analysis

Sr and Nd isotopic ratios of both whole rock and phlogopite minerals were measured using a Nu Plasma HR multicollector mass spectrometer (MC-ICP-MS) at the State Key Laboratory of Geological Process and Mineral Resources, China University of Geosciences (Beijing). During the course of our analyses, NBS987 standard yielded ^87^Sr/^86^Sr = 0.710258 ± 0.000012 (2σ), comparable with its long-term measured value of ^87^Sr/^86^Sr = 0.710274 ± 0.000021 (2σ, N = 61). The measured ^87^Sr/^86^Sr value of BHVO-2 standard is 0.703455 ± 0.000011 (2σ), and its ^143^Nd/^144^Nd value is ^143^Nd/^144^Nd = 0.512960 ± 0.000017 (2σ). Alfa Nd (An ultrapure single elemental standard solution from the China Iron and Steel Research Institute) was analyzed and used as the in-house reference. The long-term measured average value of Alfa Nd is ^143^Nd/^144^Nd = 0.512423 ± 0.000024 (2σ, N = 58). The Alfa Nd for this study is 0.512418 ± 0.000013 (2σ). Whole rock Pb isotopic compositions were determined using a Finnigan Triton TI TIMS at the State Key Laboratory for Mineral Deposits Research, Nanjing University. Approximately 100 mg of powder were dissolved in HNO_3_ + HCl mixture, and separated using a column with 50 μm of AG 1-X8 anionic resin. The extracted Pb was purified in a second column. Approximately 100 ng Pb was loaded onto a single rhenium filament using the silica-gel technique. Analytical reproducibilities of 0.01% (2σ) for ^206^Pb/^204^Pb, 0.01% for ^207^Pb/^204^Pb and 0.02% for ^208^Pb/^204^Pb were attained in this study. Mass fractionation corrections were made from runs of the NBS-981 standard based on the value suggested by Todt *et al*.^48^.

## Electronic supplementary material

Supplementary Info
