# Supplementary material for: Partial Melting of Subducted Sediments Produced Early Mesozoic Calc-alkaline Lamprophyres from Northern Guangxi Province, South China
Source: Sci Rep. 2017 Jul 7;7:4864. doi: 10.1038/s41598-017-05228-w (PMC5501806; doi:10.1038/s41598-017-05228-w)
Supplement: Supplementary file 1 — Supplementary Info [file 41598_2017_5228_MOESM1_ESM.pdf]

Supplementary information

Partial Melting of Subducted Sediments Produced Early Mesozoic Calc-alkaline Lamprophyres from Northern Guangxi Province, South China

Hui-Min Su[1], Shao-Yong Jiang[1, 2]*, Dong-Yang Zhang[1], and Xiang-Ke Wu[3]

[1]*State Key Laboratory of Geological Processes and Mineral Resources, Collaborative Innovation Center for Exploration of Strategic Mineral Resources, Faculty of Earth Resources, China University of Geosciences, Wuhan 430074, China.*

[2]*State Key Laboratory for Mineral Deposits Research, Department of Earth Sciences, Nanjing University, Nanjing 210093, China.*

[3]*Geological Survey Institute of Guangxi, Guangxi Bureau of Geology and Mineral Prospecting and Exploitation, Nanning 530023, China.*

*****Corresponding author. Shao-Yong Jiang, State Key Laboratory of Geological Processes and Mineral Resources, China University of Geosciences, Wuhan, 430074, Hubei Province, China. Tel.: +86 13952003223. *E-mail addresses*: shyjiang@cug.edu.cn (S.Y. Jiang).

Geological Setting and Petrography

The South China Block is composed of two major Precambrian continental blocks: the Yangtze Block to the northwest, and the Cathaysia Block to the southeast[1-4] (Supplementary Figure 1a). It is commonly considered that the two blocks amalgamated along the Jiangshan–Shaoxing and Pingxiang–Yushan sutures during the late Neoproterozoic time[5-9]. The Yangtze Block has an Archean to Paleoproterozoic basement, whereas the Cathaysia Block is characterized by a Paleo- to Meso-Proterozoic basement[10-13]. The upper sequences of the two blocks mainly consist of lower Paleozoic marine and upper Paleozoic continental strata and Mesozoic continental sequences[14].

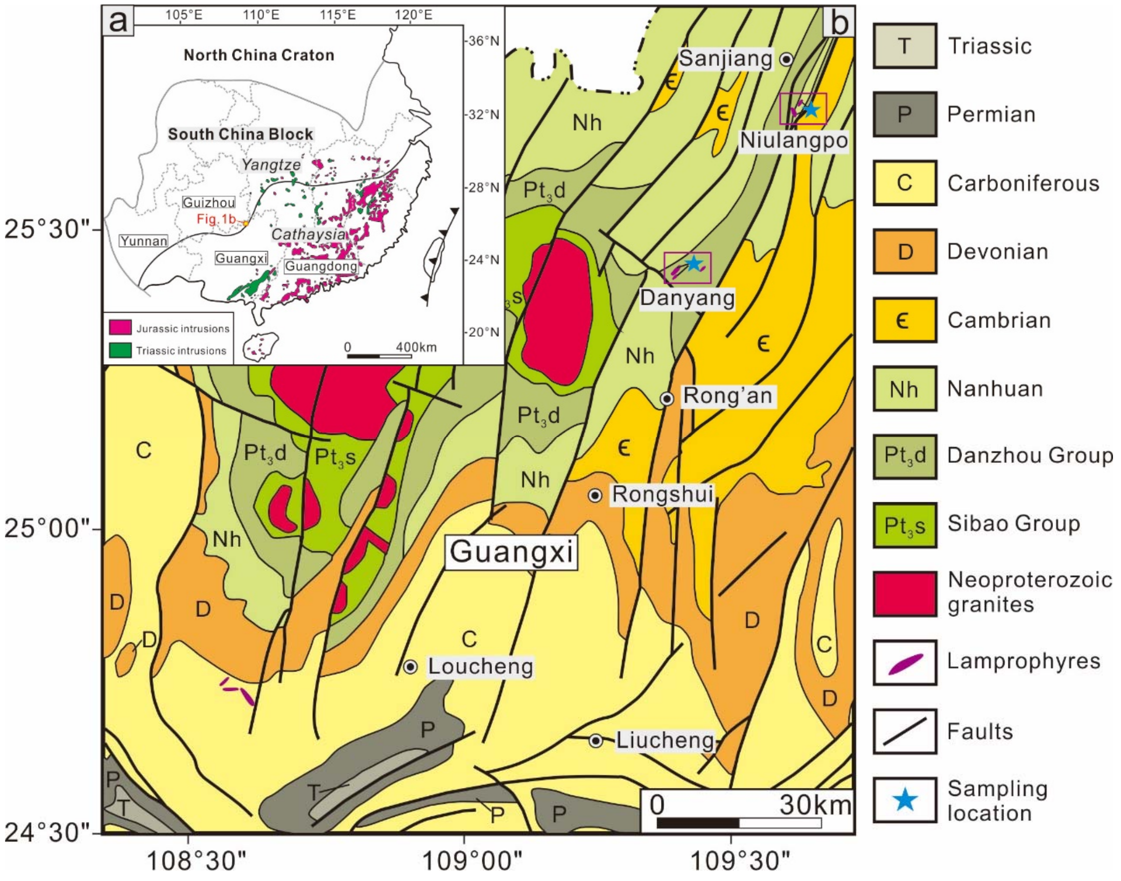

Supplementary Figure 1 (a) Simplified geologic map of the South China Block showing the distribution of Mesozoic magmatism. Modified from ref. 15; (b) Geologic map of the northern Guangxi Province showing distribution of the lamprophyre dykes. Modified from ref. 16.

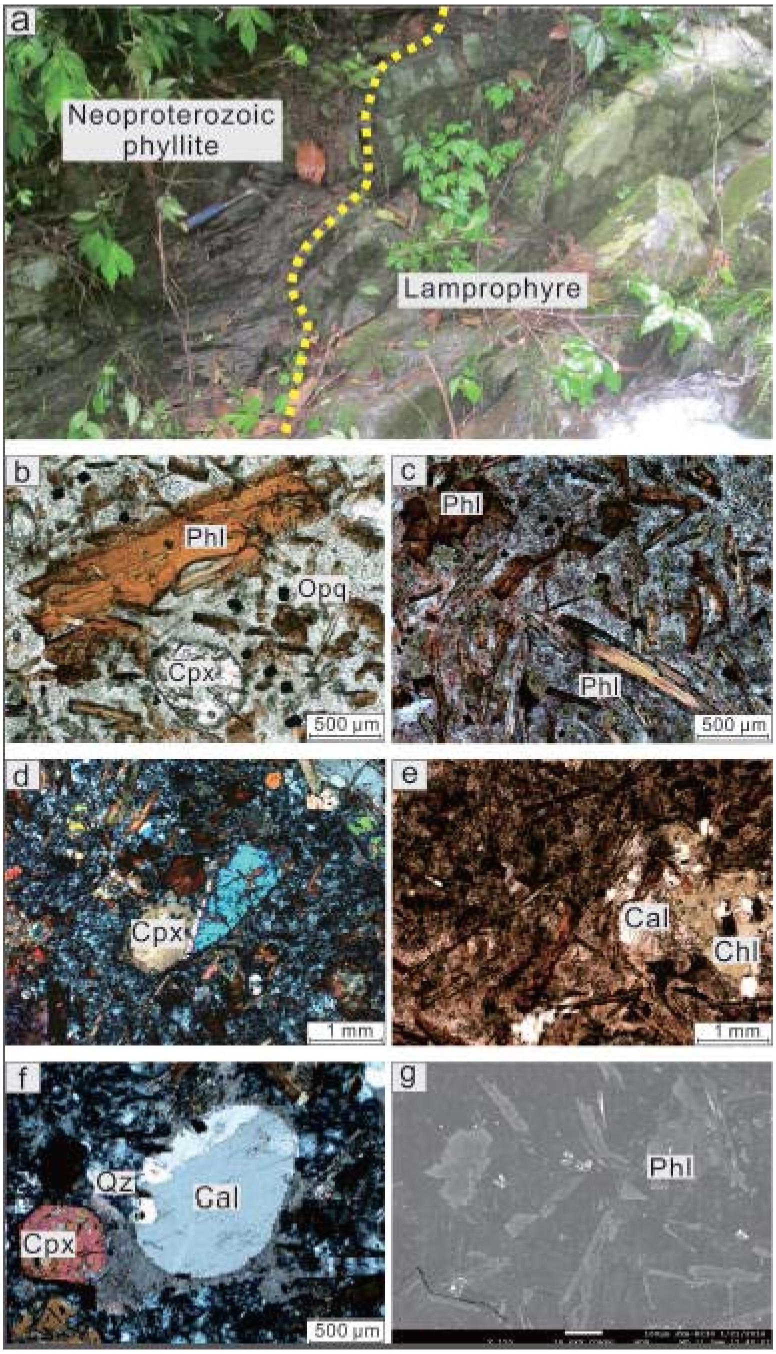

Supplementary Figure 2 Photographs, photomicrographs and back-scattered images of the studied lamprophyres from the northern Guangxi Province. Photos are taken by Hui-Min Su. (a) the Danyang lamprophyre intruded into the Neoproterozoic phyllite; (b) the Danyang lamprophyre showing phenocrysts of clinopyroxene and phlogopite; (c) the Niulangpo lamprophyre showing phenocrysts of phlogopite; (d) clinopyroxene phenocrysts from the Danyang showing twinning texture; (e) the groundmass replaced by chlorite and calcite in the Niulangpo lamprophyre; (f) spherical carbonate ocelli; (g) back-scattered images of phlogopite phenocrysts from Niulangpo showing optical zonation. Mineral

abbreviations: Phl-phlogopite; Cpx-clinopyroxene; Qz-quartz; Cal-calcite; Opq-opaque mineral.

Early Mesozoic magmatic activity in South China Block is limited both in spatial distribution and scale[17-20]. Our recent investigations have identified several Early Mesozoic lamprophyric dykes in the northern Guangxi Province, southeastern Yangtze Block. The lamprophyres outcrop in two main regions: Sanjiang-Rongshui and Luocheng (Supplementary Figure 1b), occurring as dykes with a dominant NNE trend, parallel to the major faults in this area. These dykes vary from 0.8 to 10 m in width and from 20 to 200 m in length, and intrude Precambrian to Carboniferous metasedimentary and metamorphic rocks (Supplementary Figure 2a).

The samples studied in this paper were collected from the Danyang and Niulangpo localities in the Sanjiang-Rongshui region (Supplementary Figure 1b). All the lamprophyres are holocrystalline and display typical porphyritic textures with some different mineralogical features. The Danyang samples contain predominantly mica and subordinate clinopyroxene phenocrysts (Supplementary Figure 2b). The Niulangpo samples are characterized by presence of mica as the only mineral in the phenocrystal phase (Supplementary Figure 2c). In both cases, these euhedral mafic phenocrysts comprise up to 40% of the rock and are immersed in a microcrystalline groundmass of mainly alkali-feldspar and biotite, with accessory quartz, apatite, turbid carbonate and opaque minerals. Clinopyroxene phenocrysts in the Danyang samples are prismatic, euhedral to subhedral, and exhibit twinning in places (Supplementary Figure 2d). Mica phenocrysts of various sizes are euhedral with a pale brown core and narrow dark brown rim. A darkened rim is especially evident in pseudohexagonal mica phenocrysts. Skeletal and kink-banded mica also occur. Acicular apatite is a common accessory groundmass mineral, and occurs as inclusion in both clinopyroxene and mica phenocrysts. Occasionally, barite occurs as small, interstitial crystals. The Danyang lamprophyre samples are

relatively fresh without obvious alteration, whereas the Niulangpo samples show slight alteration in the matrix with chlorite, carbonate and quartz (Supplementary Figure 2e). Spherical ocelli of mainly coarse-grained calcite are present in the samples and may reflect liquid immiscibility during emplacement (Supplementary Figure 2f)[21,22]. A particular feature of the Niulangpo lamprophyre is the presence of quartz xenocrysts ranging in size from 1.4 to 0.4 mm.

Supplementary Discussion

Genetic type of the lamprophyres

According to Rock[23], lamprophyres are divided into five different groups: calc-alkaline lamprophyre, alkaline lamprophyre, ultramafic lamprophyre, lamproite and kimberlitic lamprophyre. Some previous studies suggested that the lamprophyres in the study area show lamproitic affinity[24]. On the K_2O-SiO_2 classification diagram (Supplementary Figure 3), the Danyang lamprophyres fall within the overlapping fields of calc-alkaline lamprophyre and lamproite, whereas the Niulangpo samples plot near the boundary between them. Obviously, it is difficult to identify the type of the lamprophyres studied here on the basis of their whole-rock compositions. Mineral chemistry may be more effective in distinguishing different lamprophyres[23]. On the Al-Mg-Fe^{2+} and Al_2O_3-$Mg^{\#}$ diagrams (Supplementary Figure 4a, b), phlogopite phenocrysts from the Danyang and Niulangpo lamprophyres all fall within the domain of the calc-alkaline lamprophyre.

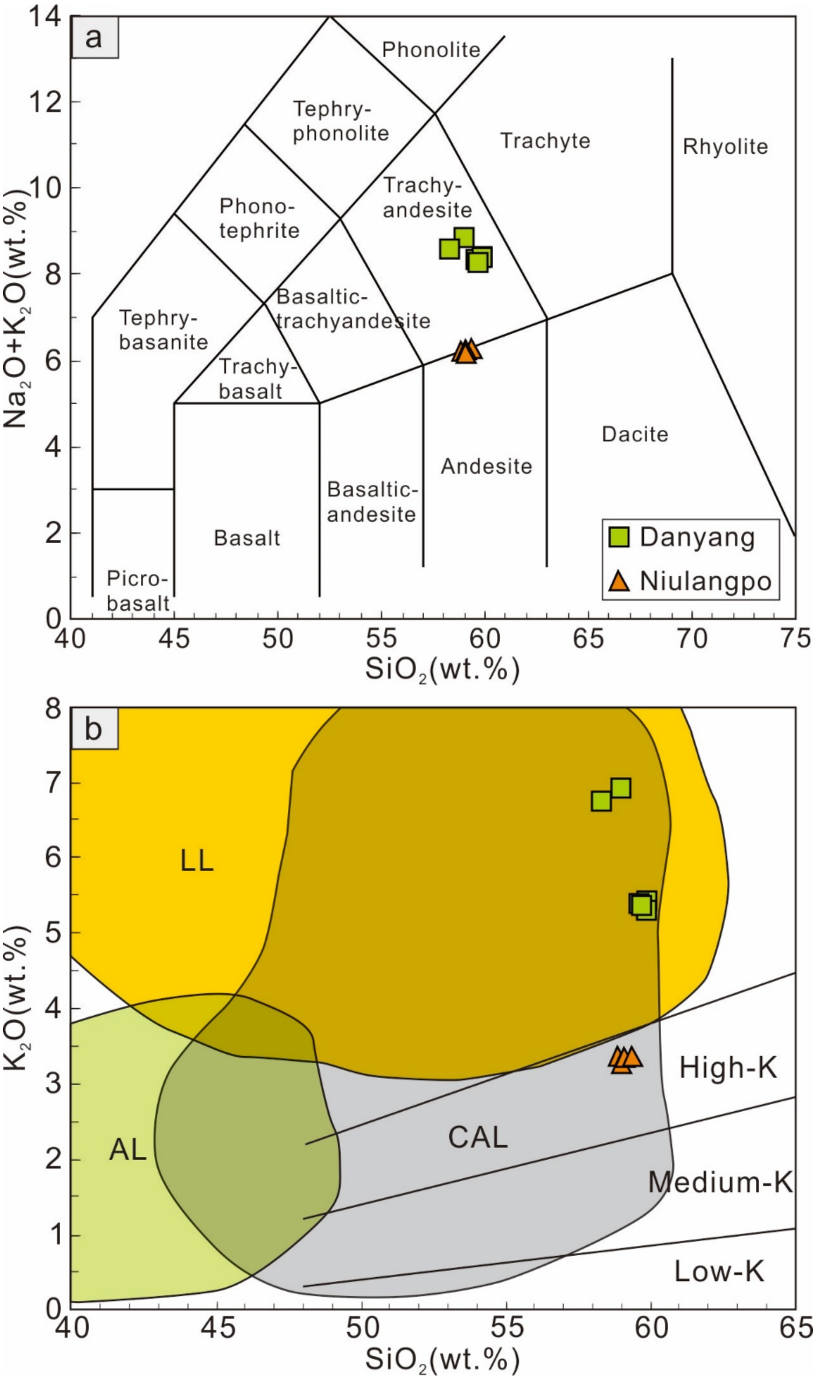

Supplementary Figure 3 (a) TAS (total alkali versus silica) diagram (after ref. 25) and (b) K_2O-SiO_2 diagram (after ref. 26) for the Danyang and Niulangpo lamprophyres. Fields for different types of lamprophyre after ref. 23. CAL-Calc-alkaline lamprophyre; AL-Alkaline lamprophyre; LL-Lamproite.

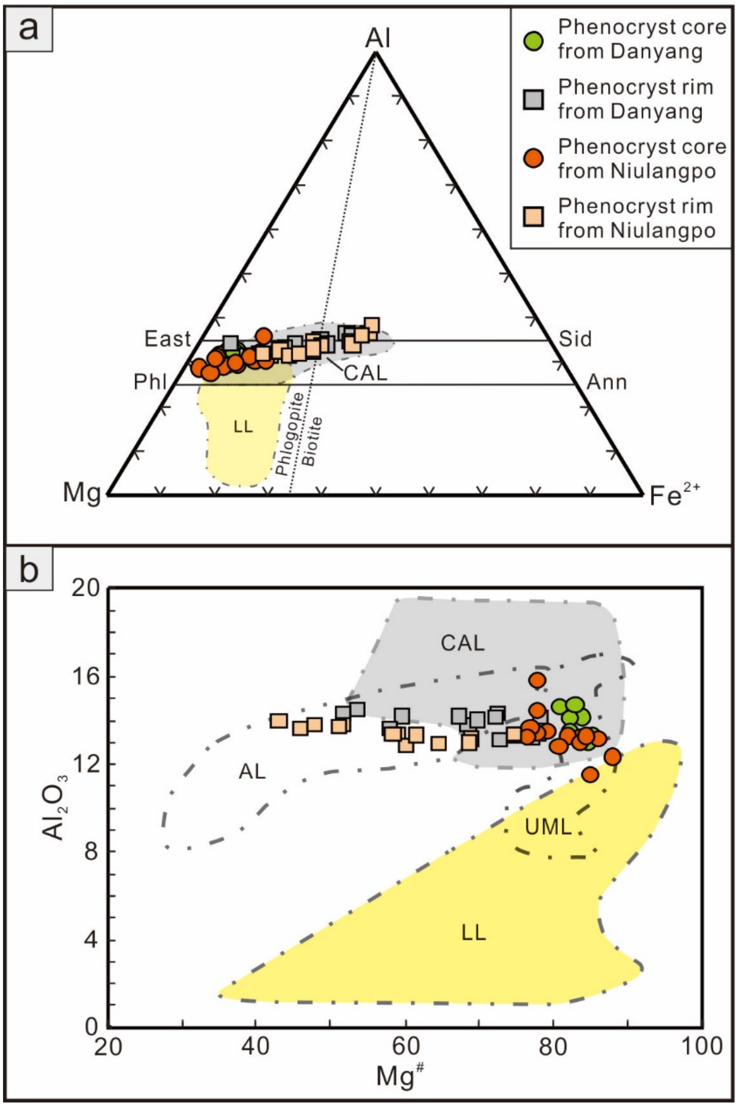

Supplementary Figure 4 (a) Al-Mg-Fe^{2+} diagram and (b) Al_2O_3-$Mg^{\#}$ diagram (after ref. 23) for the micas.

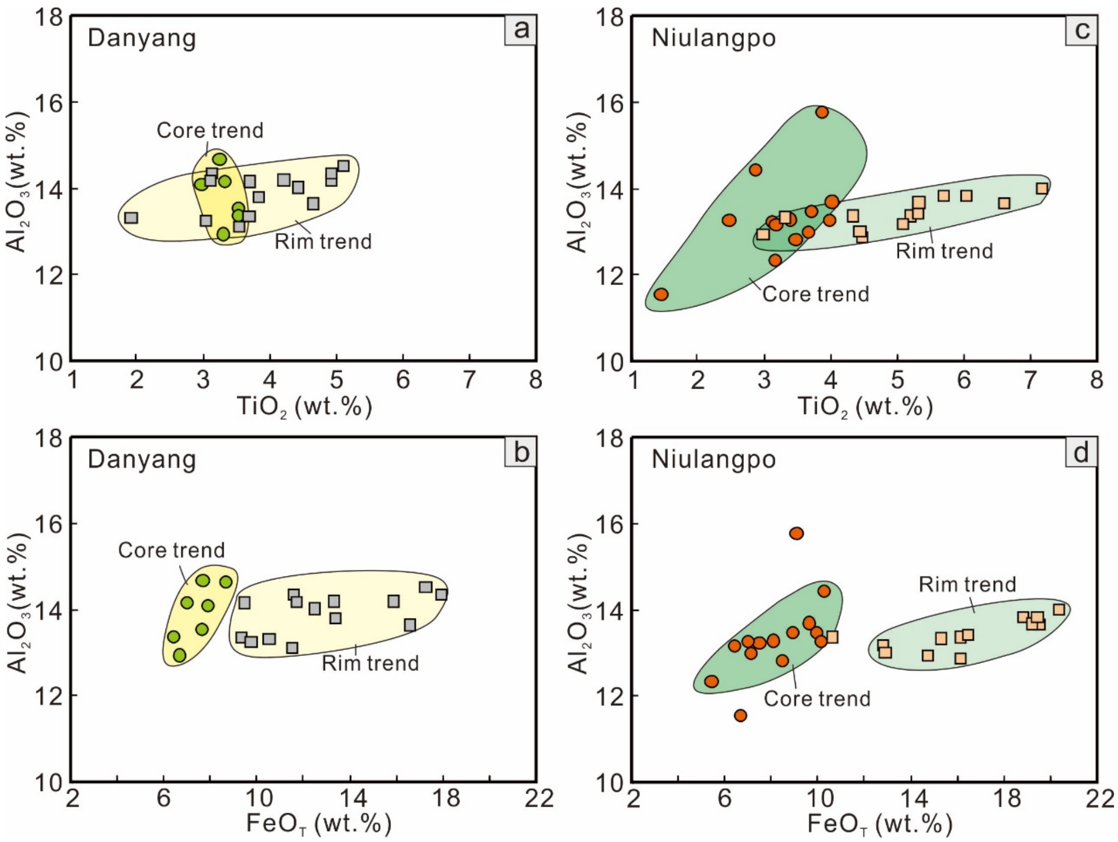

Supplementary Figure 5 Diagrams for Al_2O_3 versus TiO_2 (a, c), and Al_2O_3 versus FeO_T (b, d) for the micas.

Moreover, one of the key features of major element variations in micas from calc-alkaline lamprophyres is their increasing Al_2O_3 content with increasing TiO_2 and FeO_t contents[27]. This trend is an important criterion for distinguishing mica in calc-alkaline lamprophyre and lamproite[28]. The phlogopite phenocryst and phlogopite-biotite groundmass exhibit marked differences between Al_2O_3, and TiO_2 and FeO_t and have different trends between the rims and cores (Supplementary Figure 5a, b, c, d). Integrating these characters of petrography and mineral chemistry, the studied lamprophyres are calc-alkaline lamprophyre rather than lamproite and could be further classified as minettes according to the criteria of IUGS[25].

Alteration and crustal contamination

Secondary alteration of minerals is common in lamprophyres and could affect the final geochemical and isotopic signatures[23]. Loss on ignition (LOI) is

a useful parameter to evaluate the degree of alteration. For lamprophyres in general, samples containing <5% LOI are usually considered to be fresh[23,29]. The Danyang lamprophyres, with LOI values varying from 3.04% to 4.03%, are very fresh and do not display signs of alteration in either hand specimen or thin section. The Niulangpo samples have relatively higher LOI values ranging from 6.76% to 7.36%. Minor degrees of alteration have occurred as shown by petrographical observation. However, most trace elements, especially alkalis and alkali earth elements (such as K, Rb and Sr), do not display strong variations, suggesting that alteration did not influence the whole rock compositions greatly.

The petrographic evidence of zircon xenocrysts in both the Danyang and Niulangpo samples, and quartz xenocrysts in the Niulangpo samples, emphasizes the importance of crustal assimilation during magma ascent. However, several lines of evidence argue against a strong interaction between magmas and crustal rocks. First, the high $Mg^{\#}$ (>70) and compatible trace element contents (Cr, Co and Ni) of the lamprophyres are inconsistent with bulk assimilation of continental crust. Second, the concentrations of incompatible elements in these rocks are, in many cases, much higher than those of crustal rocks and sediments[30,31], leaving them insensitive to crustal contributions. Finally, the $\varepsilon_{Nd}(t)$ values of phlogopite separates are indistinguishable, within analytical error, from the whole rock values (Supplementary Table 3), implying that the enriched Sr-Nd isotope compositions cannot be attributed to melt contamination at crustal levels. In summary, crustal contamination is not important in the genesis of the lamprophyres.

References

1 Charvet, J., Shu, L. S., Shi, Y. S., Guo, L. Z. & Faure, M. The building of South China: collision of Yangzi and Cathaysia blocks, problems and tentative answers. *J. Southe. Asian Earth Sci.* **13,** 223-235 (1996).

2 Chen, J. F., Foland, K. A., Xing, F. M., Xu, X. & Zhou, T. X. Magmatism along the southeast margin of the Yangtze Block: Precambrian collision of the Yangtze and Cathysia blocks of

China. *Geology* **19,** 815-818 (1991).

3 Li, X. H., Zhao, J. X., McCulloch, M. T., Zhou, G. Q. & Xing, F. M. Geochemical and Sm-Nd isotopic study of Neoproterozoic ophiolites from southeastern China: petrogenesis and tectonic implications. *Precambrian Res.* **81,** 129-144 (1997).

4 Zhao, G. C. & Cawood, P. A. Precambrian geology of China. *Precambrian Res.* **222–223,** 13-54 (2012).

5 Wang, X. L. *et al.* Geochemical zonation across a Neoproterozoic orogenic belt: Isotopic evidence from granitoids and metasedimentary rocks of the Jiangnan orogen, China. *Precambrian Res.* **242,** 154–171 (2014).

6 Yao, J. L., Shu, L. S., Santosh, M. & Zhao, G. C. Neoproterozoic arc-related mafic–ultramafic rocks and syn-collision granite from the western segment of the Jiangnan Orogen, South China: Constraints on the Neoproterozoic assembly of the Yangtze and Cathaysia Blocks. *Precambrian Res.* **243,** 39-62 (2014).

7 Zhou, J. C., Wang, X. L. & Qiu, J. S. Geochronology of Neoproterozoic mafic rocks and sandstones from northeastern Guizhou, South China: Coeval arc magmatism and sedimentation. *Precambrian Res.* **170,** 27-42 (2009).

8 Zhou, M. F., Yan, D. P., Kennedy, A. K., Li, Y. & Ding, J. SHRIMP U–Pb zircon geochronological and geochemical evidence for Neoproterozoic arc-magmatism along the western margin of the Yangtze Block, South China. *Earth Planet. Sci. Lett.* **196,** 51-67 (2002).

9 Zhou, M. F. *et al.* The Yanbian Terrane (Southern Sichuan Province, SW China): A Neoproterozoic arc assemblage in the western margin of the Yangtze Block. *Precambrian Res.* **144,** 19-38 (2006).

10 Chen, J. F. & Jahn, B. Crustal evolution of southeastern China: Nd and Sr isotopic evidence. *Tectonophysics* **284,** 101-133 (1998).

11 Zheng, J. P. *et al.* Widespread Archean basement beneath the Yangtze craton. *Geology* **34,** 417-420 (2006).

12Yu, J. H. *et al.* Components and episodic growth of Precambrian crust in the Cathaysia Block, South China: Evidence from U–Pb ages and Hf isotopes of zircons in Neoproterozoic sediments. *Precambrian Res.* **181,** 97-114 (2010).

13 Yu, J. H. *et al.* Formation history and protolith characteristics of granulite facies metamorphic rock in Central Cathaysia deduced from U-Pb and Lu-Hf isotopic studies of single zircon grains. *Chin. Sci. Bull.* **50,** 2080-2089 (2005).

14 Wang, Y. J., Fan, W. M., Cawood, P. A. & Li, S. Z. Sr–Nd–Pb isotopic constraints on multiple mantle domains for Mesozoic mafic rocks beneath the South China Block hinterland. *Lithos* **106,** 297-308 (2008).

15 Liu, C. Z., Liu, Z. C., Wu, F. Y. & Chu, Z. Y. Mesozoic accretion of juvenile sub-continental lithospheric mantle beneath South China and its implications: Geochemical and Re–Os isotopic results from Ningyuan mantle xenoliths. *Chem. Geol.* **291,** 186-198 (2012).

16 Wang, X. L. *et al.* LA-ICP-MS U-Pb zircon geochronology of the Neoproterozoic igneous rocks from Northern Guangxi, South China: Implications for tectonic evolution. *Precambrian Res.* **145,** 111-130 (2006).

17 Cai, Y., Lu, J., Ma, D., Huang, H., Zhang, H. & Zhang, R. The late Triassic Dengfuxian A-type granite, Hunan Province: age, petrogenesis, and implications for understanding the late Indosinian tectonic transition in South China. *Int. Geol. Rev*. **57**, 428-445 (2015).

18 Li, W. Y., Ma, C. Q., Liu, Y. Y. & Robinson, P. T. Discovery of the Indosinian aluminum A-type granite in Zhejiang Province and its geological significance. *Sci. Chin. (Ser. D): Earth Sci.* **55**, 13-25 (2012).

19 Zhao, K. D., Jiang, S. Y., Chen, W. F., Chen, P. R. & Ling, H. F. Zircon U-Pb chronology and elemental and Sr-Nd-Hf isotope geochemistry of two Triassic A-type granites in South China: implication for petrogenesis and Indosinian transtensional tectonism. *Lithos* **160-161**, 292-306 (2013)

20 Wang, Y. J., Fan, W. M., Zhang, G. W. & Zhang, Y. H. Phanerozoic tectonics of the South China Block: Key observations and controversies. *Gondwana Res.* **23,** 1273-1305 (2013).

21 Woodard, J., Kietäväinen, R. & Eklund, O. Svecofennian post-collisional shoshonitic lamprophyres at the margin of the Karelia Craton: Implications for mantle metasomatism. *Lithos* **205,** 379-393 (2014).

22 Ferguson, J. & Currie, K. L. Evidence of liquid immiscibility in alkaline ultrabasic dikes at Callander Bay, Ontario. *J. Petrol.* **12(3),** 561-585 (1971).

23 Rock, N. M. S. The nature and origin of lamprophyres: an overview. *Geol. Soc., Lond. Spec. Publ.* **30,** 191-226 (1987).

24 Wang, X. Y., Peng, S. B., Wu, X. K. & Lu, G. Discovery of lamproites in the northern Guangxi and its prospecting significance. *Geol. Sci. Tech. Infor.* **3,** 93 (2013) (in Chinese).

25 Le Maitre, R. W. *Igneous Rocks: A Classification and Glossary of Terms: Recommendations of the International Union of Geological Sciences Subcommission on the Systematics of Igneous Rocks.* Blackwell Scientific Publictions 1-236 (1989).

26 Peccerillo, A. & Taylor, S. R. Geochemistry of eocene calc-alkaline volcanic rocks from the Kastamonu area, Northern Turkey. *Contrib Mineral. Petr.* **58,** 63-81 (1976).

27 Prelević, D., Foley, S. F., Cvetković, V. & Romer, R. L. Origin of minette by mixing of lamproite and dacite magmas in Veliki Majdan, Serbia. *J. Petrol.* **45,** 759-792 (2004).

28 Mitchell, R. H. & Bergman, S. C. *Petrology of Lamproites.* Springer US 1-447 (1991).

29 Wooley, A. R. *et al.* Classification of lamprophyres, lamproites, kimberlites, and the kalsilitic, melilitic, and leucitic rocks. *Can. Mineral.* **34,** 175-186 (1996).

30 Taylor, S. R. & McLennan, S. M. *The Continental Crust: its Composition and Evolution.* Blackwell Scientific Publications 1-312 (1985).

31 Plank, T. & Langmuir, C. H. The chemical composition of subducting sediment and its consequences for the crust and mantle. *Chem. Geol.* **145,** 325-394 (1998).

33 Peccerillo, A. & Taylor, S. R. Geochemistry of eocene calc-alkaline volcanic rocks from the Kastamonu area, Northern Turkey. *Contrib Mineral. Petr.* **58,** 63-81 (1976).

Supplementary Tables

Supplementary Table 1 Ar-Ar isotope dating results for phlogopite from the Danyang and Niulangpo lamprophyres.

Supplementary Table 2 Major and trace element analyses of the Danyang and Niulangpo lamprophyres.

Supplementary Table 3 Sr and Nd isotopes for selected whole rock and phlogopite samples from the Danyang and Niulangpo lamprophyres.

Supplementary Table 4 Whole rock Pb isotope data for the Danyang and Niulangpo lamprophyres.

Supplementary Table 5 Electron microprobe analyses of micas from the Danyang and Niulangpo lamprophyres.

Supplementary Table 1 $^{40}Ar/^{39}Ar$ isotope dating results for phlogopite from the Danyang and Niulangpo lamprophyres

T (℃)	$^{39}/^{40}Ar$	error	$^{39}/^{36}Ar$	error	$^{36}/^{40}Ar$	error	$^{40}Ar^{*}$ (%)	^{39}Ar (Mol)	$^{40}Ar^{*}/^{39}Ar$	error	Age (Ma)	1σ
DY-06; J=0.003569												
900	9.94E-02	2.89E-03	6.74E+01	5.63E+00	1.48E-03	1.20E-04	56.36	5.88E-15	5.67E+00	3.45E-01	36	2
950	3.48E-02	3.90E-04	9.81E+01	4.29E+01	3.50E-04	1.50E-04	89.51	4.08E-15	2.58E+01	1.31E+00	159	8
1000	2.79E-02	8.00E-05	1.29E+03	3.72E+03	2.00E-05	6.00E-05	99.34	1.32E-14	3.56E+01	6.61E-01	216	4
1050	2.76E-02	9.00E-05	7.37E+02	4.59E+02	4.00E-05	2.00E-05	98.87	1.89E-14	3.58E+01	2.60E-01	217	2
1100	2.78E-02	1.00E-04	1.09E+03	1.90E+03	3.00E-05	4.00E-05	99.23	2.03E-14	3.57E+01	4.74E-01	216	3
1150	2.82E-02	1.00E-05	5.86E+03	5.91E+04	0.00E+00	5.00E-05	99.84	2.43E-14	3.55E+01	5.08E-01	215	3
1200	2.83E-02	7.00E-05	2.78E+04	1.39E+06	0.00E+00	5.00E-05	99.95	2.77E-14	3.53E+01	5.34E-01	214	3
1250	2.82E-02	8.00E-05	8.74E+03	1.36E+04	0.00E+00	0.00E+00	99.89	3.49E-14	3.55E+01	1.16E-01	215	1
1300	2.80E-02	3.00E-05	1.64E+03	5.38E+02	2.00E-05	1.00E-05	99.48	4.03E-14	3.55E+01	6.05E-02	215	1
1350	2.84E-02	1.20E-04	2.79E+03	1.17E+04	1.00E-05	4.00E-05	99.68	1.49E-14	3.51E+01	4.53E-01	213	3
1400	2.78E-02	1.50E-04	7.09E+02	2.86E+02	4.00E-05	2.00E-05	98.82	2.06E-14	3.55E+01	2.12E-01	215	2
1450	2.73E-02	8.00E-05	3.09E+02	2.88E+01	9.00E-05	1.00E-05	97.38	1.85E-14	3.57E+01	1.07E-01	216	1
1500	2.73E-02	1.80E-04	2.66E+02	5.70E+01	1.00E-04	2.00E-05	96.95	1.52E-14	3.56E+01	3.26E-01	216	2
1550	2.78E-02	8.00E-05	1.32E+03	1.72E+02	2.00E-05	0.00E+00	99.36	1.25E-14	3.57E+01	1.06E-01	217	1
NLP-04; J=0.003471												
800	5.53E-02	1.30E-04	5.47E+01	2.36E+00	1.01E-03	4.00E-05	70.13	6.50E-15	1.27E+01	2.27E-01	78	1
850	4.80E-02	2.20E-04	8.57E+01	1.53E+00	5.60E-04	1.00E-05	83.41	3.36E-14	1.74E+01	5.93E-02	106	1
900	3.98E-02	2.10E-04	1.11E+02	6.64E+00	3.60E-04	2.00E-05	89.37	3.79E-14	2.24E+01	1.52E-01	135	1
950	3.46E-02	4.00E-05	1.59E+02	1.51E+01	2.20E-04	2.00E-05	93.54	3.96E-14	2.70E+01	1.80E-01	162	1
1000	3.13E-02	6.00E-05	1.87E+02	2.76E+00	1.70E-04	0.00E+00	95.05	3.83E-14	3.04E+01	6.04E-02	181	1
1050	3.05E-02	5.00E-05	2.61E+02	8.86E+00	1.20E-04	0.00E+00	96.54	3.22E-14	3.17E+01	4.31E-02	188	1
1100	2.82E-02	1.40E-04	3.33E+02	6.18E+01	8.00E-05	2.00E-05	97.48	2.59E-14	3.46E+01	2.14E-01	204	2
1150	2.62E-02	9.00E-05	2.33E+02	1.18E+01	1.10E-04	1.00E-05	96.66	3.72E-14	3.70E+01	1.23E-01	218	1
1200	2.64E-02	9.00E-05	3.09E+02	4.37E+01	9.00E-05	1.00E-05	97.46	3.88E-14	3.69E+01	1.80E-01	217	2
1250	2.69E-02	7.00E-05	1.43E+03	7.35E+02	2.00E-05	1.00E-05	99.43	4.32E-14	3.69E+01	1.40E-01	218	1
1300	2.71E-02	4.00E-05	1.32E+04	6.96E+04	0.00E+00	1.00E-05	99.92	4.53E-14	3.68E+01	1.21E-01	217	1
1350	2.73E-02	8.00E-05	4.20E+03	9.29E+03	1.00E-05	1.00E-05	99.79	3.78E-14	3.66E+01	1.82E-01	216	2
1400	2.74E-02	7.00E-05	5.29E+03	3.18E+03	1.00E-05	0.00E+00	99.83	2.59E-14	3.65E+01	6.97E-02	215	1
1450	2.70E-02	1.50E-04	9.06E+02	1.25E+02	3.00E-05	0.00E+00	99.10	1.32E-14	3.67E+01	1.71E-01	216	2
1500	2.63E-02	1.05E-03	3.01E+02	1.49E+03	9.00E-05	4.30E-04	97.40	1.76E-15	3.70E+01	5.04E+00	218	28

Supplementary Table 2 Major and trace element analyses of the Danyang and Niulangpo lamprophyres

		Danyang						Niulangpo			
Sample		DY-01	DY-02	DY-03	DY-04	DY-05	DY-06	NLP-01	NLP-02	NLP-03	NLP-04
Major elements											
SiO_2	%	56.70	55.49	56.74	55.96	56.29	56.92	53.80	53.93	53.92	53.97
TiO_2	%	0.69	0.93	0.68	0.94	0.70	0.69	1.04	1.07	1.05	1.09
Al2O3	%	14.02	12.92	13.98	13.17	13.93	14.10	11.43	11.64	11.52	11.62
TFe_2O_3	%	5.13	5.67	5.11	5.50	5.20	5.17	5.63	5.73	5.63	5.74
FeO	%	2.50	2.78	2.60	2.71	2.53	2.93	2.50	2.68	2.50	2.96
MnO	%	0.12	0.10	0.10	0.11	0.11	0.10	0.10	0.10	0.10	0.10
MgO	%	6.13	7.51	6.03	6.69	6.08	6.07	7.02	7.22	7.00	6.94
CaO	%	4.20	4.11	4.03	3.80	4.11	3.94	5.98	5.53	5.52	6.08
Na_2O	%	2.76	1.76	2.93	1.84	2.80	2.86	2.60	2.75	2.68	2.62
K_2O	%	5.12	6.44	5.04	6.57	5.10	5.17	3.05	2.99	3.05	3.12
P_2O_5	%	0.48	0.62	0.47	0.62	0.48	0.48	0.70	0.71	0.70	0.72
LOI	%	3.81	3.24	3.76	3.29	4.03	3.53	7.36	7.11	7.12	6.76
Total	%	98.88	98.48	98.58	98.19	98.55	98.70	98.43	98.48	98.01	98.43
$Mg^{\#}$		70	72	70	71	70	70	71	71	71	71
trace elements											
Li	ppm	34.5	54.2	30.4	56.1	31.7	30.8	108	108.	118	112
Be	ppm	5.33	6.33	4.95	8.14	5.22	4.56	6.75	6.93	7.71	6.83
Sc	ppm	13.3	16.0	13.4	15.2	13.5	12.4	15.7	16.3	17.6	16.6
Tl	ppm	1.40	1.97	1.43	2.03	1.45	1.42	1.06	1.06	1.19	1.10
V	ppm	105	118	103	120	106	106.	118	126	130	126
Cr	ppm	252	347	250	320	254	247	396	426	440	431
Co	ppm	24.8	30.3	28.0	28.5	26.0	25.3	29.5	31.9	35.1	30.8
Ni	ppm	186	283	275	227	198	179	250	270	381	247
Cu	ppm	49.7	88.4	67.9	91.9	50.2	47.5	104	86.2	97.4	88.3
Zn	ppm	53.5	59.7	49.9	58.3	52.6	51.3	59.0	59.6	63.9	61.6
Ga	ppm	18.1	17.9	18.1	18.2	18.2	17.9	15.3	16.47	17.7	17.1
Rb	ppm	207	308	212	296	215	203	140	144	159	155
Sr	ppm	694	495	583	648	637	609	616	491	640	739
Y	ppm	16.8	18.9	17.2	18.1	17.2	17.6	16.7	17.8	19.2	17.5
Zr	ppm	433	598	445	646	433	441	699	748	799	752
Nb	ppm	17.7	20.7	18.3	21.9	17.8	17.9	21.7	23.2	24.8	23.4
Cs	ppm	8.57	10.1	8.78	14.6	8.06	8.05	5.38	5.97	6.24	6.04
Ba	ppm	1725	3199	2331	3626	3414	3716	3802	2599	3446	3685
La	ppm	121	126	123	131	121	124	102	112	114	108.
Ce	ppm	220	240	226	253	222	227	213	228	237	231
Pr	ppm	24.0	27.5	24.7	28.8	24.2	24.7	25.9	28.3	29.0	27.8
Nd	ppm	76.0	88.6	77.9	90.4	76.5	78.7	88.0	98.1	100.5	96.4
Sm	ppm	10.2	12.2	10.6	12.8	10.5	10.3	12.0	13.0	14.1	13.9
Eu	ppm	1.72	1.98	1.78	2.06	1.76	1.73	2.12	2.28	2.42	2.28
Gd	ppm	6.69	7.92	6.90	8.22	6.98	7.12	8.30	8.95	9.43	8.91
Tb	ppm	0.74	0.84	0.75	0.87	0.76	0.77	0.87	0.93	0.98	0.93
Dy	ppm	3.38	3.72	3.44	3.84	3.58	3.63	3.71	3.93	4.23	4.07
Ho	ppm	0.65	0.68	0.65	0.69	0.65	0.68	0.68	0.72	0.77	0.72
Er	ppm	1.71	1.76	1.72	1.81	1.79	1.81	1.76	1.77	1.92	1.83
Tm	ppm	0.25	0.25	0.25	0.26	0.25	0.26	0.24	0.25	0.27	0.25
Yb	ppm	1.54	1.59	1.60	1.64	1.57	1.62	1.53	1.57	1.72	1.59
Lu	ppm	0.23	0.24	0.24	0.24	0.24	0.25	0.23	0.23	0.26	0.24
Hf	ppm	13.4	18.7	14.0	20.3	13.7	14.0	22.6	23.7	25.9	24.6
Ta	ppm	1.29	1.41	1.46	1.44	1.34	1.18	1.44	1.56	1.72	1.52
Pb	ppm	80.6	94.9	74.8	84.2	71.9	81.6	98.9	118.7	125.8	107.8
Bi	ppm	0.31	0.31	0.34	0.22	0.29	0.27	1.45	1.33	1.50	1.29
Th	ppm	74.2	88.0	77.9	95.2	77.0	79.0	85.0	88.8	96.5	92.0
U	ppm	8.73	10.2	9.32	10.8	9.10	9.36	10.5	11.0	12.0	11.2
ΣREE	ppm	468	513	480	536	472	483	460	501	517	499
$(La/Yb)_N$		56.2	56.9	55.4	57.3	55.1	55.1	47.8	51.2	47.5	48.8
Eu/Eu*		0.63	0.62	0.64	0.61	0.63	0.61	0.65	0.65	0.64	0.63

Note: $Mg^{\#}$ is 100*MgO/(MgO+FeO_T) in molar proportions; $(La/Yb)_N$=La_N/Yb_N; Eu/Eu*= $Eu_N / \sqrt{Sm_N * Gd_N}$, where N means Chondrite-normalized value. Analytical uncertainties are 0.5%-1.5% for major elements according to their abundances, and are better than 5% for trace elements.

Supplementary Table 3 Sr and Nd isotopic compositions for selected whole rock and phlogopite samples from the Danyang and Niulangpo lamprophyres

Sample	Rock/Mineral	Age (Ma)	Rb (ppm)	Sr (ppm)	$^{87}Rb/^{86}Sr$	$^{87}Sr/^{86}Sr$	2σ	$(^{87}Sr/^{86}Sr)_i$	Sm (ppm)	Nd (ppm)	$^{147}Sm/^{144}Nd$	$^{143}Nd/^{144}Nd$	2σ	$(^{143}Nd/^{144}Nd)_i$	$\varepsilon_{Nd}(0)$	$\varepsilon_{Nd}(t)$	T_{DM} (Ma)
DY-02	Whole Rock	216	308	495	1.806	0.72438	0.00001	0.71883	12.2	88.6	0.0830	0.51203	0.00001	0.51191	-11.9	**-8.76.**	1308
DY-05	Whole Rock	216	215	637	0.978	0.72220	0.00001	0.71919	10.5	76.5	0.0828	0.51202	0.00001	0.51190	-12.1	**-9.00**	1321
DY-06	Phlogopite	216	454	156	8.422	0.74215	0.00002	0.71628	2.89	22.8	0.0765	0.51199	0.00001	0.51188	-12.7	**-9.41**	1294
NLP-02	Whole Rock	217	144	491	0.851	0.72144	0.00001	0.71881	13.0	98.1	0.0799	0.51204	0.00001	0.51192	-11.7	**-8.48**	1268
NLP-04a	Phlogopite	217	562	198	8.228	0.74039	0.00002	0.71499	2.53	18.3	0.0834	0.51205	0.00001	0.51193	-11.4	**-8.29**	1284
NLP-04b	Phlogopite	217	562	198	8.228	0.74067	0.00003	0.71527	2.53	18.3	0.0834	0.51206	0.00002	0.51195	-11.2	**-8.05**	1270

Supplementary Table 4 Whole rock Pb isotope data for the Danyang and Niulangpo lamprophyres

Sample	t (Ma)	Pb (ppm)	Th (ppm)	U (ppm)	$^{206}Pb/^{204}Pb$	2σ	$^{207}Pb/^{204}Pb$	2σ	$^{208}Pb/^{204}Pb$	2σ	$(^{206}Pb/^{204}Pb)_i$	$(^{207}Pb/^{204}Pb)_i$	$(^{208}Pb/^{204}Pb)_i$
DY-01	216	81	74	9	18.4902	0.0004	15.7139	0.0004	39.3097	0.0012	18.2528	15.7019	38.6521
DY-02	216	95	88	10	18.5041	0.0004	15.7133	0.0004	39.3246	0.0011	18.2678	15.7014	38.6620
DY-03	216	75	78	9	18.5522	0.0005	15.7184	0.0004	39.4281	0.0013	18.2780	15.7046	38.6817
DY-04	216	84	95	11	18.5763	0.0004	15.7202	0.0004	39.4920	0.0010	18.2926	15.7059	38.6803
DY-05	216	72	77	9	18.5597	0.0005	15.7195	0.0004	39.4431	0.0011	18.2812	15.7054	38.6751
DY-06	216	82	79	9	18.5195	0.0005	15.7208	0.0005	39.3655	0.0013	18.2677	15.7081	38.6724
NLP-01	217	99	85	10	18.5402	0.0005	15.7220	0.0004	39.2797	0.0012	18.3062	15.7102	38.6621
NLP-02	217	119	89	11	18.5132	0.0004	15.7207	0.0003	39.2259	0.0011	18.3094	15.7104	38.6888
NLP-03	217	126	97	12	18.5263	0.0004	15.7218	0.0004	39.2452	0.0013	18.3155	15.7112	38.6942
NLP-04	217	108	92	11	18.5413	0.0005	15.7294	0.0004	39.2905	0.0012	18.3131	15.7179	38.6775

Supplementary Table 5 Electron microprobe analyses of micas from the Danyang and Niulangpo lamprophyres

Sample	DY-01				DY-02							DY-04
Location	1c	1r	2c	2r	1c	1r	2c	2r	3r	4r	5r	1c
SiO_2	39.64	38.08	38.73	37.93	38.84	41.35	39.03	38.88	38.13	34.52	36.77	39.54
TiO_2	3.32	3.12	2.39	3.10	2.97	1.91	3.54	3.69	3.54	5.11	4.92	3.54
Al_2O_3	14.17	14.38	14.65	14.18	14.11	13.34	13.54	14.17	13.12	14.54	14.21	13.39
FeO^t	7.05	11.59	8.65	11.69	7.88	10.54	7.65	9.46	11.50	17.25	15.91	6.42
MnO	0.03	0.03	0.04	0.06	0.02	0.09	0.00	0.02	0.05	0.15	0.09	0.00
MgO	20.40	17.09	20.53	17.18	20.47	17.80	19.90	18.96	17.20	11.23	13.30	20.56
CaO	0.02	0.02	0.07	0.00	0.03	0.07	0.00	0.07	0.01	0.02	0.03	0.06
Na_2O	0.68	0.47	0.50	0.48	0.59	0.40	0.42	0.50	0.46	0.55	0.48	0.51
K_2O	9.67	9.59	8.28	9.38	9.47	6.17	9.95	9.87	9.83	9.46	9.48	10.10
P_2O_5	0.02	0.00	0.00	0.00	0.00	0.00	0.02	0.00	0.00	0.00	0.00	0.00
NiO	0.03	0.04	0.05	0.02	0.05	0.04	0.01	0.07	0.00	0.00	0.03	0.05
Cr_2O_3	0.31	0.00	0.13	0.02	0.02	0.21	0.05	0.19	0.00	0.00	0.00	0.10
F	0.80	-	0.83	-	0.74	0.68	1.10	-	0.85	0.33	0.42	1.08
Cl	0.10	-	0.10	-	0.12	0.08	0.05	-	0.07	0.11	0.04	0.09
Total	96.21	94.41	94.94	94.05	95.32	92.67	95.25	95.87	94.75	93.27	95.68	95.41
Si	5.67	5.66	5.60	5.66	5.62	6.06	5.65	5.65	5.66	5.39	5.52	5.69
Al^{IV}	2.33	2.34	2.40	2.34	2.38	1.94	2.31	2.35	2.30	2.61	2.48	2.27
Al^{VI}	0.06	0.18	0.10	0.15	0.02	0.36	0.00	0.08	0.00	0.06	0.04	0.00
Ti	0.36	0.35	0.26	0.35	0.32	0.21	0.39	0.40	0.39	0.60	0.56	0.38
Fe^{3+}	0.44	0.29	0.48	0.29	0.37	0.95	0.47	0.28	0.41	0.40	0.46	0.47
Fe^{2+}	0.41	1.15	0.57	1.17	0.58	0.34	0.45	0.87	1.02	1.85	1.54	0.30
Mn	0.00	0.00	0.00	0.01	0.00	0.01	0.00	0.00	0.01	0.02	0.01	0.00
Mg	4.35	3.79	4.43	3.82	4.41	3.89	4.30	4.11	3.81	2.61	2.98	4.41
Ca	0.00	0.00	0.01	0.00	0.01	0.01	0.00	0.01	0.00	0.00	0.00	0.01
Na	0.19	0.14	0.14	0.14	0.16	0.11	0.12	0.14	0.13	0.17	0.14	0.14
K	1.76	1.82	1.53	1.79	1.75	1.15	1.84	1.83	1.86	1.88	1.82	1.85
Total	15.56	15.71	15.52	15.71	15.63	15.05	15.53	15.72	15.59	15.60	15.54	15.53
$Mg^{\#}$	84	72	81	72	82	75	82	78	73	53	60	85

Table 5 (*Continued*)

Sample	DY-04									NLP-01		
Location	1r	2c	2r	3c	3r	4r	5r	6r	7r	1c	1r	2c
SiO_2	38.80	39.75	38.52	39.54	38.96	36.93	36.97	37.07	35.42	39.16	34.76	40.95
TiO_2	3.84	3.31	3.69	3.24	3.04	4.21	4.41	4.64	4.92	3.12	6.60	3.15
Al_2O_3	13.82	12.96	13.37	14.71	13.25	14.21	14.04	13.67	14.35	13.23	13.68	12.36
FeO^t	13.39	6.70	9.36	7.68	9.75	13.29	12.48	16.56	17.92	7.49	19.52	5.43
MnO	0.06	0.00	0.04	0.04	0.10	0.08	0.04	0.16	0.27	0.04	0.23	0.06
MgO	16.33	20.59	18.78	20.91	18.54	15.32	16.16	12.86	10.91	21.34	9.40	22.47
CaO	0.04	0.04	0.04	0.00	0.02	0.08	0.03	0.01	0.02	0.05	0.10	0.01
Na_2O	0.40	0.63	0.43	0.45	0.47	0.51	0.68	0.48	0.58	0.41	0.66	0.35
K_2O	9.65	10.06	9.76	9.63	9.90	9.49	9.59	9.63	9.52	10.13	9.46	10.21
P_2O_5	0.00	0.00	0.02	0.01	0.00	0.06	0.00	0.00	0.01	0.02	0.01	0.04
NiO	0.02	0.06	0.00	0.08	0.08	0.03	0.05	0.01	0.00	0.09	0.00	0.03
Cr_2O_3	0.05	0.10	0.02	0.05	0.04	0.04	0.04	0.02	0.00	0.07	0.00	0.34
F	-	1.08	1.17	0.72	1.00	0.62	0.85	0.58	0.27	1.34	0.56	1.38
Cl	-	0.17	0.05	0.01	0.04	0.10	0.10	0.05	0.12	0.01	0.14	0.02
Total	96.40	95.45	95.26	97.04	95.17	94.94	95.44	95.73	94.31	96.49	95.10	96.82
Si	5.69	5.73	5.62	5.60	5.71	5.53	5.49	5.59	5.48	5.61	5.39	5.79
Al^{IV}	2.31	2.20	2.30	2.40	2.29	2.47	2.46	2.41	2.52	2.23	2.50	2.06
Al^{VI}	0.08	0.00	0.00	0.06	0.00	0.03	0.00	0.01	0.09	0.00	0.00	0.00
Ti	0.42	0.36	0.41	0.35	0.34	0.47	0.49	0.53	0.57	0.34	0.77	0.34
Fe^{3+}	0.35	0.44	0.48	0.41	0.43	0.43	0.42	0.47	0.41	0.39	0.53	0.50
Fe^{2+}	1.29	0.36	0.66	0.50	0.76	1.23	1.13	1.61	1.90	0.51	2.00	0.15
Mn	0.01	0.00	0.01	0.00	0.01	0.01	0.01	0.02	0.04	0.00	0.03	0.01
Mg	3.57	4.43	4.09	4.42	4.05	3.42	3.58	2.89	2.51	4.56	2.17	4.73
Ca	0.01	0.01	0.01	0.00	0.00	0.01	0.01	0.00	0.00	0.01	0.02	0.00
Na	0.11	0.18	0.12	0.12	0.13	0.15	0.20	0.14	0.17	0.11	0.20	0.10
K	1.80	1.85	1.82	1.74	1.85	1.81	1.82	1.85	1.88	1.85	1.87	1.84
Total	15.65	15.56	15.52	15.59	15.57	15.57	15.58	15.53	15.59	15.61	15.47	15.50
$Mg^{\#}$	68	85	78	83	77	67	70	58	52	83	46	88

Table 5 (*Continued*)

Sample	NLP-01					NLP-03						
Location	2r	3c	3r	4c	4r	1c	1r	2c	2r	3c	3r	4c
SiO_2	35.27	38.08	36.94	39.94	35.83	41.31	36.78	39.58	37.34	38.57	38.48	38.20
TiO_2	5.70	3.71	5.20	3.71	7.16	3.17	4.46	3.38	5.09	4.01	4.32	3.87
Al_2O_3	13.86	13.50	13.38	13.49	14.00	13.18	12.87	13.31	13.19	13.72	13.38	15.79
FeO^t	18.85	8.94	16.13	9.95	20.34	6.41	16.11	8.08	12.79	9.63	10.63	9.09
MnO	0.17	0.02	0.15	0.08	0.14	0.06	0.17	0.05	0.00	0.07	0.04	0.02
MgO	11.41	19.00	13.11	19.49	8.70	21.94	13.77	20.77	15.86	18.07	17.54	17.82
CaO	0.01	0.01	0.04	0.02	0.03	0.01	0.10	0.03	0.02	0.02	0.01	0.02
Na_2O	0.40	0.37	0.38	0.28	0.36	0.36	0.55	0.30	0.40	0.29	0.40	0.34
K_2O	9.09	10.01	8.87	9.83	9.13	10.29	9.14	9.48	9.51	10.03	9.09	10.25
P_2O_5	0.03	0.00	0.00	0.02	0.01	0.00	0.00	0.00	0.05	0.04	0.00	0.00
NiO	0.02	0.11	0.03	0.03	0.00	0.05	0.08	0.04	0.00	0.05	0.05	0.03
Cr_2O_3	0.01	0.00	0.02	0.10	0.02	0.20	0.00	0.14	0.00	0.16	0.00	0.13
F	0.53	-	0.85	-	0.36	-	0.97	-	0.95	-	-	-
Cl	0.04	-	0.08	-	0.07	-	0.20	-	0.04	-	-	-
Total	95.37	93.74	95.17	96.93	96.14	96.98	95.19	95.16	95.24	94.65	93.95	95.57
Si	5.40	5.65	5.57	5.73	5.47	5.84	5.57	5.73	5.55	5.69	5.70	5.56
Al^{IV}	2.50	2.35	2.38	2.27	2.52	2.16	2.30	2.27	2.31	2.31	2.30	2.44
Al^{VI}	0.00	0.02	0.00	0.01	0.00	0.03	0.00	0.00	0.00	0.07	0.04	0.27
Ti	0.66	0.41	0.59	0.40	0.82	0.34	0.51	0.37	0.57	0.44	0.48	0.42
Fe^{3+}	0.50	0.25	0.65	0.33	0.71	0.29	0.52	0.32	0.54	0.34	0.44	0.34
Fe^{2+}	1.92	0.86	1.38	0.86	1.89	0.46	1.52	0.66	1.04	0.85	0.88	0.77
Mn	0.02	0.00	0.02	0.01	0.02	0.01	0.02	0.01	0.00	0.01	0.00	0.00
Mg	2.61	4.21	2.94	4.17	1.98	4.62	3.11	4.48	3.51	3.97	3.88	3.87
Ca	0.00	0.00	0.01	0.00	0.00	0.00	0.02	0.00	0.00	0.00	0.00	0.00
Na	0.12	0.11	0.11	0.08	0.11	0.10	0.16	0.08	0.11	0.08	0.12	0.10
K	1.78	1.90	1.71	1.80	1.78	1.85	1.76	1.75	1.80	1.89	1.72	1.90
Total	15.50	15.75	15.35	15.67	15.29	15.71	15.48	15.68	15.46	15.66	15.56	15.66
$Mg^{\#}$	52	79	59	78	43	86	60	82	69	77	75	78

Table 5 (*Continued*)

Sample	NLP-03	NLP-04										
Location	4r	1c	1r	2c	2r	3c	3r	4c	4r	4c	5r	6c
SiO_2	37.29	37.81	37.31	41.56	35.57	40.26	35.99	37.03	38.33	39.15	36.09	39.89
TiO_2	5.32	3.98	4.43	1.45	6.03	3.65	5.30	2.86	2.97	3.46	3.30	2.47
Al_2O_3	13.70	13.27	13.03	11.55	13.86	13.01	13.45	14.45	12.96	12.83	13.36	13.29
FeO^t	19.17	10.13	12.89	6.66	19.43	7.16	16.45	10.29	14.69	8.48	15.28	7.02
MnO	0.20	0.09	0.11	0.05	0.13	0.08	0.15	0.09	0.09	0.06	0.15	0.02
MgO	11.38	18.41	15.91	21.29	10.04	20.48	13.03	20.44	15.04	20.00	13.81	21.12
CaO	0.04	0.02	0.00	0.04	0.02	0.04	0.02	0.09	0.03	0.04	0.02	0.05
Na_2O	0.34	0.35	0.34	0.31	0.32	0.31	0.35	0.30	0.35	0.48	0.35	0.43
K_2O	9.47	9.90	9.38	10.07	9.11	10.04	9.20	7.65	9.69	10.16	9.25	8.82
P_2O_5	0.00	0.01	0.00	0.00	0.01	0.00	0.02	0.00	0.02	0.00	0.00	0.00
NiO	0.00	0.03	0.01	0.04	0.08	0.03	0.00	0.06	0.01	0.00	0.00	0.00
Cr_2O_3	0.00	0.03	0.05	0.11	0.00	0.20	0.01	0.06	0.01	0.08	0.00	0.35
F	-	-	-	1.26	-	1.37	0.72	1.00	1.03	1.37	0.84	1.21
Cl	-	-	-	0.01	-	0.01	0.01	0.01	0.02	0.08	0.02	0.11
Total	96.90	94.03	93.45	94.38	94.59	96.64	94.70	94.33	95.24	96.18	92.46	94.77
Si	5.60	5.63	5.65	6.02	5.50	5.73	5.48	5.43	5.73	5.65	5.60	5.76
Al^{IV}	2.40	2.33	2.33	1.97	2.50	2.18	2.41	2.50	2.27	2.18	2.40	2.24
Al^{VI}	0.03	0.00	0.00	0.00	0.03	0.00	0.00	0.00	0.02	0.00	0.04	0.02
Ti	0.60	0.45	0.50	0.16	0.70	0.39	0.61	0.32	0.33	0.38	0.39	0.27
Fe^{3+}	0.46	0.25	0.36	0.48	0.52	0.57	0.53	0.47	0.48	0.44	0.44	0.57
Fe^{2+}	1.95	1.01	1.27	0.33	1.99	0.28	1.57	0.79	1.36	0.58	1.54	0.27
Mn	0.03	0.01	0.01	0.01	0.02	0.01	0.02	0.01	0.01	0.01	0.02	0.00
Mg	2.55	4.09	3.59	4.60	2.32	4.35	2.96	4.47	3.36	4.30	3.19	4.54
Ca	0.01	0.00	0.00	0.01	0.00	0.01	0.00	0.01	0.01	0.01	0.00	0.01
Na	0.10	0.10	0.10	0.09	0.10	0.09	0.10	0.09	0.10	0.13	0.10	0.12
K	1.82	1.88	1.81	1.86	1.80	1.82	1.79	1.43	1.85	1.87	1.83	1.62
Total	15.54	15.75	15.64	15.52	15.48	15.43	15.47	15.53	15.52	15.56	15.56	15.43
$Mg^{\#}$	51	76	69	85	48	83	58	78	64	81	61	84

Cation calculated on the basis of 22 O. $Mg^{\#}$: $100*Mg/(Mg+Fe^{2+})$. The accuracy is 1%-5% depending on the abundance of the element.
